# Gene–Diet Interactions on Metabolic Disease-Related Outcomes in Southeast Asian Populations: A Systematic Review

**DOI:** 10.3390/nu15132948

**Published:** 2023-06-29

**Authors:** Padmini Sekar, Eduard Flores Ventura, Anto Cordelia T. A. Dhanapal, Eddy Seong Guan Cheah, Annaletchumy Loganathan, Phoon Lee Quen, Mahenderan Appukutty, Nurpudji Astuti Taslim, Hardinsyah Hardinsyah, Mohd Fairulnizal Md Noh, Julie A Lovegrove, Ian Givens, Karani Santhanakrishnan Vimaleswaran

**Affiliations:** 1 Hugh Sinclair Unit of Human Nutrition, Department of Food and Nutritional Sciences, Institute for Cardiovascular and Metabolic Research (ICMR), University of Reading, Reading RG6 6DZ, UK; p.sekar@pgr.reading.ac.uk (P.S.); eduard.flores.ventura@hotmail.com (E.F.V.); j.a.lovegrove@reading.ac.uk (J.A.L.); 2 Centre for Biomedical and Nutrition Research, Universiti Tunku Abdul Rahman, Jalan Universiti, Bandar Barat, Kampar 31900, Malaysia; antoc@utar.edu.my (A.C.T.A.D.); cheahsg@utar.edu.my (E.S.G.C.); annal@utar.edu.my (A.L.); phoonlq@utar.edu.my (P.L.Q.); 3 Faculty of Sports Science and Recreation, Universiti Teknologi MARA, Shah Alam 40450, Malaysia; mahen@uitm.edu.my; 4 Nutrition Society of Malaysia, Jalan PJS 1/48 off Jalan Klang Lama, Petaling Jaya 46150, Malaysia; 5 Clinical Nutrition, Faculty of Medicine, Hasanuddin University, Makassar 90245, Indonesia; pudji_taslim@yahoo.com; 6 Department of Community Nutrition, Faculty of Human Ecology, IPB University, Bogor 16680, Indonesia; hardinsyah2010@gmail.com; 7 Institute for Medical Research, National Institutes of Health, Jalan Setia Murni U13/52, Seksyen U13 Setia Alam, Shah Alam 40170, Malaysia; fairulnizal@moh.gov.my; 8 Institute for Food, Nutrition and Health (IFNH), University of Reading, Reading RG6 6AH, UK; d.i.givens@reading.ac.uk

**Keywords:** systematic review, nutrigenetics, Southeast Asia, genetics, gene–diet interaction, dietary intake, obesity, diabetes, metabolic disease

## Abstract

Diabetes and obesity are chronic diseases that are a burden to low- and middle-income countries. We conducted this systematic review to understand gene–diet interactions affecting the Southeast Asian population’s risk of obesity and diabetes. The literature search was performed on Google Scholar and MEDLINE (PubMed) search engines independently by four reviewers who evaluated the eligibility of articles based on inclusion criteria. Out of 19,031 articles, 20 articles examining gene–diet interactions on obesity and/or diabetes-related traits met the inclusion criteria. Three (Malaysia, Indonesia, and Singapore) out of eleven Association of Southeast Asian Nations (ASEAN) countries have conducted studies on gene–diet interactions on obesity and diabetes. From the 20 selected articles, the most common interactions were observed between macronutrients and genetic risk score (GRS) on metabolic disease-related traits in the Malay, Chinese, and Indian ethnicities. Overall, we identified 29 significant gene–diet interactions in the Southeast Asian population. The results of this systematic review demonstrate ethnic-specific gene–nutrient interactions on metabolic-disease-related traits in the Southeast Asian population. This is the first systematic review to explore gene–diet interactions on obesity and diabetes in the Southeast Asian population and further research using larger sample sizes is required for better understanding and framing nutrigenetic approaches for personalized nutrition.

## 1. Introduction

Metabolic diseases such as obesity and diabetes are now considered epidemics rapidly spreading across developed and developing countries affecting both sexes, age, ethnicities, and socioeconomic groups [1]. This, in turn, has shown to compromise the quality of life that leads to potentially life-threatening conditions such as cancers, cardiovascular diseases, musculoskeletal disorders, and hypertension [2]. According to the 2021 reports from the World Health Organisation (WHO), worldwide obesity has tripled since 1975, with over 650 million obese adults and more than 340 million children and adolescents who are either overweight or obese [3]. By 2022, diabetes reports from WHO indicate that more than 422 million people are diabetic with rising prevalence in low-middle income group countries (LMIC) compared to developed countries [4]. Projected trends also show that diabetes and obesity are rapidly growing and will affect nearly two-thirds of the Southeast (SE) Asian population by the end of 2030, placing a burden on rural and low socioeconomic groups [5,6]. Recent reports from the Association of Southeast Asian Nations (ASEAN) show that the tripling rate of undernutrition has not improved and that obesity and diabetes are now a double burden for these countries [7,8]. 

Understanding gene–nutrient interactions provide insights regarding nutritional, genetic, and biochemical determinants to better understand complex interactions between environmental factors (including diet) and genes relevant to metabolic health and dis-eases [9,10]. Several studies have also reported the importance of physical activity and nutrient intake which potentially interact with genetic predispositions of a disease that promote the progression and pathogenesis of metabolic diseases [11]. Many studies have also reported the influence of certain gene–diet interactions on metabolic disease-related traits and emphasized the importance of a healthy lifestyle that may modify the outcome of the disease or its related parameters [10,11,12,13,14,15]. A better comprehension of the relationship between genes and diet is key to making correlations between nutrition and wellness, thereby allowing for specific nutritional suggestions that are tailored to individuals or genetic subgroups. This strategy presents an appropriate public health approach [9].

The increasing prevalence of diabetes and obesity in SE Asia can be understood by the nutrition transition phenomenon, environment multiplier theory, and the thrifty gene hypothesis [16,17]. These theories provide an understanding of the dietary shift from traditional high-carbohydrate, low-fat diets towards high-energy diets (high saturated fat, sugars, and salt), and the role of inherited genetic predispositions in over-nutrition-related diseases. Dietary factors can affect the outcome of a disease and there are ethnic-specific genetic variations that influence the mechanism of these nutrient interactions [2]. Furthermore, lifestyle/dietary factors could influence genetic predispositions of metabolic disorders, especially obesity, and diabetes [18,19,20,21], making nutrigenetics research a necessity in ethnically diverse populations such as SE Asia. Nutrition science along with a better understanding of nutrigenetics in different ethnic groups is important to improvise personal and societal health [2,20]. This would ultimately add to the efforts of implementing precision nutrition specific to the populations [22,23]. Hence, this systematic review examines gene–diet interactions on metabolic disease-related (diabetes and obesity) outcomes in the 11 SE Asian countries (Brunei, Burma (Myanmar), Cambodia, Timor-Leste, Indonesia, Laos, Malaysia, the Philippines, Singapore, Thailand, and Vietnam) that share sufficient socio-demographic and cultural similarities.

## 2. Materials and Methods

### 2.1. Study Identification and Source Strategy

To identify studies involving gene–diet interactions on metabolic disease-related outcomes, a literature search was undertaken until February 2023 using MEDLINE (via PubMed), and Google Scholar search engines (Supplementary Table S1). The reference lists of the included papers and independent search strings used by the researchers were examined until saturation. In PubMed, extensive search was performed using the search string: (polymorphism OR gene OR SNP OR single nucleotide polymorphism OR genetic variation OR genetic variant OR GRS OR genetic risk score OR PRS OR polygenic risk score) AND (“gene–diet interaction” OR “diet-gene interaction” OR SNP-diet interaction OR diet-SNP interaction OR “gene–nutrient interaction” OR “nutrient-gene interaction” OR “gene-lifestyle inter-action” OR “gene-environment interaction”) AND (carbohydrate OR protein OR fat OR fiber OR sugar OR SFA OR saturated fat OR monounsaturated fat OR polyunsaturated fat OR MUFA OR PUFA OR diet OR B12 OR vitamin D OR amino acids OR polyphenols OR egg intake OR caffeine intake OR green tea OR alcohol intake OR meat intake OR energy intake OR food) AND (obesity OR weight OR BMI OR waist circumference OR waist hip ratio OR hip circumference OR adiposity OR metabolic diseases OR body fat OR body composition) AND (Southeast Asia OR Malay* OR Brunei* OR Burm* OR Cambodia* OR Timor* OR Indonesia* OR Laos OR Filipin* OR Philippine* OR Singapore* OR Thai* OR Vietnam*). In Google Scholar, an extensive search was performed using the search string: gene–diet interaction BMI Southeast Asia OR Malay* OR Brunei* OR Burm* OR Cambodia* OR Timor* OR Indonesia* OR Laos OR Filipin* OR Philippine* OR Singapore* OR Thai* OR Vietnam*. The literature search was restricted to studies involving human subjects only.

### 2.2. Data Extraction

The reviewers (E.V.F., P.S., A.C.T.A.D., and E.S.G.C.) ensured data consistency across the articles extracted for this study, and a narrative synthesis was conducted to compile the data sourced. Duplicate articles were eliminated using EndNote. Titles and abstracts were subjected to blind screening to assess the pre-established inclusion criteria, followed by full-text screening and discussion. The study protocol was submitted to PROSPERO (Identification number: CRD42022366475).

### 2.3. Study Selection: Inclusion and Exclusion Criteria

Related studies published in PubMed and Google Scholar in the English language were included. Only gene–nutrient interaction (nutrigenetic) studies examining the as-sociation between dietary factors and genes on diabetes and/or obesity-related outcomes were included. Eligible articles on clinical studies, multicentre studies, comparative studies, observational studies, and randomized controlled studies were included. Studies on patients, neonates, children, and pregnant women were included. The study included populations from ASEAN countries namely Brunei, Cambodia, Indonesia, Laos, Malay-sia, Myanmar, the Philippines, Singapore, Thailand, Timor-Leste, and Vietnam. Studies were excluded if they were (1) animal studies; (2) did not include gene–diet interactions; (3) the outcome was not diabetes- and/or obesity-related traits; (4) not examined in the SE Asian population; or (5) nutrigenomic studies (gene expression in response to dietary factors).

### 2.4. Data Items and Effect Measures

Obesity, diabetes, and parameters associated with anthropometric measurements and attributes were considered primary outputs of data extraction (Table 1 and Table 2). The result of interactions between the exposure (genetic and dietary factors) and outcome (obesity- or diabetes-related traits) was estimated using *p*-values extracted from the included literature. Based on the statistical output, the interactions were considered significant if the P_interaction_ values were below 0.05. In this study, a narrative synthesis was conducted to elaborate on the dietary factors, genetic variation, and disease traits.

### 2.5. Risk of Bias and Certainty Assessment

The appraisal tool for cross-sectional studies (AXIS) was used to assess the methodological quality and risk of bias (RoB) of the cross-sectional studies (Supplementary Section S1, Tables S2 and S3) [42]. The RoB in non-randomized studies of interventions (ROBINS-1) assessment tool was used for cohort, case–control, and non-randomized studies (Supplementary Section S1, Table S4) [43]. A revised Cochrane RoB tool for randomized trials (Rob2) was used for randomized control trials (Supplementary Section S1, Table S5) [41,44]. This review falls within the framework and guidelines from the synthesis without meta-analysis (SWiM) in systematic reviews [24,26].

## 3. Results and Discussion

### 3.1. Nutrigenetics Studies in Southeast Asia

**Figure 1:**
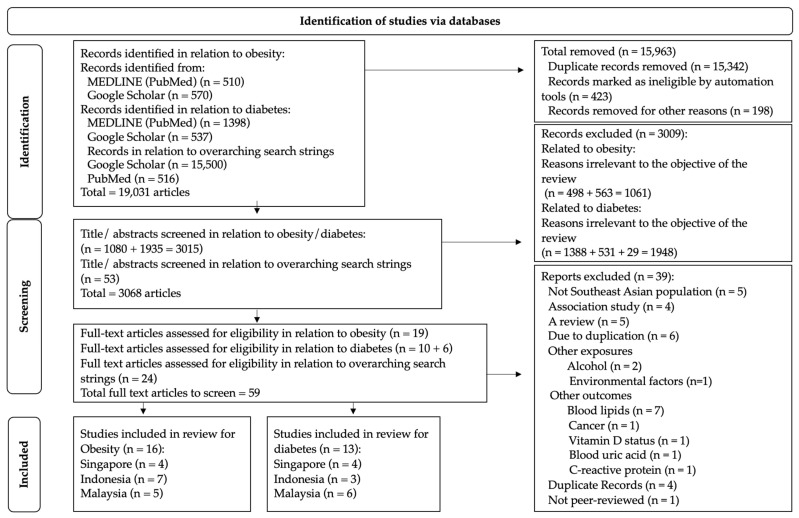
PRISMA flowchart showing the selection of articles for this study based on inclusion and exclusion criteria.

Using PubMed and Google Scholar search engines, we found 19,031 articles matching the search strings. After the full-text screening, we included a total of 20 nutrigenetic studies related to obesity- and diabetes-related parameters carried out in SE Asia. Out of this, 16 studies examined obesity-related outcomes, 13 examined diabetes-related outcomes, and 9 studies observed both obesity, and diabetes-related outcomes. Figure 1 shows the selection of the 20 studies included in this systematic review. From the included studies, only three ASEAN countries (Malaysia, Indonesia, and Singapore) conducted studies to understand gene–diet interactions on metabolic disease-related traits. 

### 3.2. Gene–Diet Interactions on Obesity-Related Outcomes in the Southeast Asian Population

**Figure 2:**
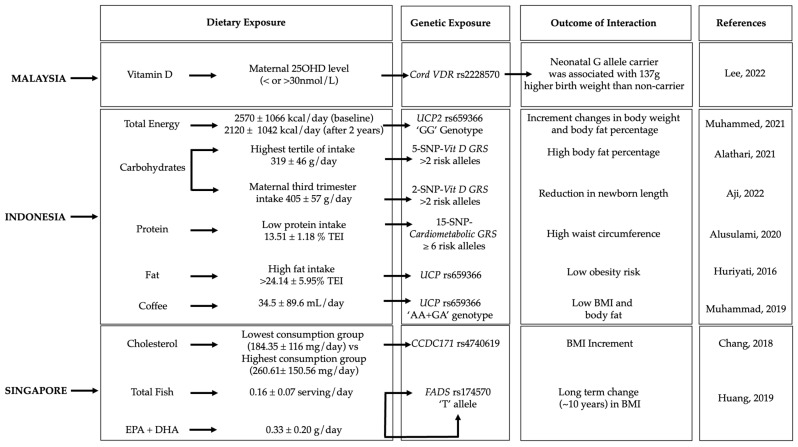
Gene–diet interactions on obesity-related traits. The figure shows the significant gene–diet interactions (*p* < 0.05) on obesity traits and the dietary factors that have influenced the risk of obesity in individuals carrying specific genetic variations in the Malaysian [25], Indonesian [12,21,29,30,31,33] and Singaporean [34,35] populations. GRS, genetic risk score; BMI, body mass index; EPA + DHA, eicosapentaenoic acid + docosahexaenoic; TEI, total energy intake; plus-minus symbol (±) indicates Standard deviation.

Gene–diet interactions on obesity-related traits were observed in five Malaysian, seven Indonesian, and four Singaporean studies (Figure 2, Table 1). 

#### 3.2.1. Malaysia

The interaction between gene variants and dietary factors on obesity-related outcomes were examined in five Malaysian studies: four cross-sectional ([24,25,27,28]) and one randomized control trial (RCT) [26]. 

One cross-sectional study (n = 200) in Chinese and Indian ethnic groups living in Malaysia reported no significant interaction between *FADS1* SNP rs174547 and linoleic acid (LA) or α-linolenic acid (ALA) on WC but showed that vegetarians with TT genotype of *FADS1* gene had higher odds of metabolic diseases and larger WC [24]. The *FADS1* gene is involved in the lipid metabolic pathway to catalyze the biosynthesis of unsaturated fatty acids and is known to play a significant role in the maintenance of triglycerol and HDL-c levels [45]. 

Another cross-sectional study in 217 individuals showed significant interaction (P_interaction_ = 0.018) between maternal vitamin D deficiency and cord *VDR* SNP rs2228570 on neonatal birth weight and an inverse association of maternal vitamin D deficiency with neonatal birth weight indicating the importance of this gene–diet interaction on fetal anthropometry [25]. Vitamin D is a secosteroid and a prohormone that plays a pivotal role in embryogenesis, calcium homeostasis, and fetal bone development and its deficiency is associated with adverse fetal and maternal outcomes [46]. This explains the importance of understanding maternal vitamin D status along with a genetic factor, here cord VDR SNP rs2228570, to examine how gene–nutrient interaction can influence neonatal birth anthropometric outcomes.

The third cross-sectional study (n = 507) in the Malay, Chinese, and Indian ethnic groups analyzed the interactions between *AGTR1* and *AGTR2* gene variants and different dietary patterns on body mass index (BMI). This study revealed no significant interactions between *AGTR1* SNP rs5186 and the dietary patterns (vegetables, fruits, soy diet (VFSD) in Malays and rice, egg, and fish diet (REFD) in Chinese) on BMI. The same study also failed to show interactions between *AGTR2* SNP rs1403543 and VFSD on BMI in Chinese women. Interestingly, this study found that the Malay and Chinese ethnic groups were at a higher risk for elevated lipids compared with the Indian ethnic group [27]. A better understanding of these genes in the context of obesity in different ethnicities is important as the renin-angiotensin system is an important regulator of adipose tissue metabolism, whole-body energy, and glucose homeostasis [47]. Previous studies have shown that overexpression of the adipose renin-angiotensin system could be associated with obesity [48].

The fourth cross-sectional study (n = 179) examined the interaction between *VEGFR2* SNP rs1870377 and meat, rice, and noodles diet on BMI in the Chinese population and showed no significant interaction [28]. Previous *in vivo* studies to understand the role of *VEGFR1* and *VEGFR2* in angiogenesis in diet-induced obesity have shown that *VEGFR2* antiangiogenic blockade may limit adipose tissue expansion in obesity [49]. Studies in larger populations of different ethnicities are needed to better understand this mechanism in the context of ethnic-specific gene–nutrient interaction as this may be a potential target for obesity prevention and treatment strategies. 

The final study is an RCT (n = 128) in the Malaysian population that observed the interaction between *FTO* (rs9930501, rs9930506, rs9932754) and *ADRB2* (rs1042713, rs1042714) gene variants and Hipcref (high-protein, calorie-restricted, high-vitamin E, and high-fiber) diet pattern on BMI, body weight, WC, WHR, fat mass, body fat percentage (BFP) and muscle mass in the Chinese, Malay, and Indian ethnicities. However, this study failed to show any significant interaction between these genes and dietary patterns on the abovementioned obesity-related parameters [26]. The *FTO* and *ADRB2* genes are widely studied specifically in relation to obesity [26,50]. An interesting finding from previous literature showed that *FTO* carriers of heterozygous risk alleles could still have a protective effect against obesity when subjected to increased physical activity and by following an appropriate weight loss regimen. Homozygous carriers of the *ADRB2* allele (G > A genotype) have been linked to lower levels of lipid mobilization, which could provide insights into creating dietary plans and obesity prevention strategies for various ethnicities [26]. Further understanding of this concept in larger groups in the SE Asian population may yield promising outcomes in the field of nutrigenetics to target obesity.

#### 3.2.2. Indonesia

Seven studies analyzed gene–diet interactions on obesity-related parameters in the Indonesian population: four cross-sectional [12,21,31,32], two prospective cohort studies [29,33] and one case–control study [30].

A cross-sectional study in 110 Minangkabau women of Indonesia showed significant interaction (P_interaction_ = 0.049) between vitamin D GRS (*DHCR7*, *CYP2R1*, *CYP24A1*, *GC*, *CASR*) and carbohydrate intake on BFP. The results indicated that participants carrying more than two risk alleles and who consumed high carbohydrate intake had significantly higher BFP than participants with less than two risk alleles. However, there was no significant interaction between metabolic-GRS (*FTO*, *TCF7L2*, *MC4R*, *KCNQ1*, *CDKN2A/B*) and carbohydrate or protein intake on obesity-related parameters [12]. Another cross-sectional study in 117 Minangkabau women showed significant interaction (P_interaction_ = 0.034) between vitamin D-associated *9-SNP-B12-GRS* and protein intake on BFP. The same study also indicated a significant interaction (P_interaction_ = 0.032) between vitamin D-associated *9-SNP-metabolic-GRS* and protein energy on WC, indicating that women consuming a low fiber diet (4.90 ± 1.00 g/day) and harboring ≥9 risk alleles for vitamin B12 deficiency had notably higher HbA1C levels than the others (P_interaction_ = 0.025) [32]. There are many mechanisms proposed to understand the role of Vitamin D levels and obesity including increased fat stores and increased vitamin D storage in adipose tissue. This also considers the lifestyle differences between obese and lean individuals along with combinatorial effects of dietary patterns to have a significant effect on obesity [51]. Further studies are needed to confirm the exact mechanism behind gene–diet interaction on these obesity-related parameters.

One study on 455 Indonesian adults from Yogyakarta examined that coffee consumption and carriers of *UCP2* SNP rs659366 *AA + GA* genotype had a negative correlation with BMI (P_interaction_ = 0.01) and body fat (kg) (P_interaction_ = 0.021) levels. The same study also showed that carriers of the *GG* genotype had no correlation with coffee consumption and obesity, indicating that gene variations and coffee intake influences obesity-related parameters [31]. Previous studies have elucidated the potential anti-obesity properties of tea and coffee [52]. Scientific evidence shows possible mechanisms of this activity via cell cycle regulation in adipocytes during adipogenesis, the effect on transcription factors involved in weight loss, and lipogenesis-related proteins [53]. However, further ethnic-specific research is needed to better understand these mechanisms as this proves to be a promising strategy to combat obesity due to the large global consumption of coffee.

Another study in 110 Minangkabau women observed that carriers of more than six risk alleles of a *15-SNP-GRS* for cardiometabolic disease and consuming low protein intake had significantly (P_interaction_ = 0.002) lower WC compared to carriers of less than six risk alleles. In addition, the study also showed a significant influence of GRS on WC and triglyceride levels through a low-protein diet specifically in Minangkabau women [21]. One prospective cohort study in the Indonesian adult population examined the interaction between two *UCP2* gene variations ((rs659366 (−866G/A) AA + GA Genotype) and rs659366 (−866G/A) GG Genotype) and dietary factors on obesity-related outcomes (body weight, BFP, waist-hip ratio (WHR)). There were no significant interactions observed between *UCP2* gene variations and any of the dietary factors on the obesity-related parameters; however, significant interaction depicting a positive correlation between *UCP2* SNP rs659366 (−866G/A) GG genotype and total energy intake on body weight change (P_interaction_ = 0.016) and BFP (P_interaction_ = 0.034) was observed. The same study also showed significant interaction (P_interaction_ = 0.040) between *UCP2* SNP rs659366 (−866G/A) GG genotype and physical activity on WHR indicating that participants with increased physical activity and the *UCP2* gene variant had lower WHR [29]. Further studies on larger populations are required to validate these results and elucidate the mechanisms.

Another study showed a significant interaction (P_interaction_ = 0.032) between *2-SNP-GRS* and carbohydrate intake on infant birth length. Pregnant women with >2 risk alleles of *VDR-GRS* and low vitamin D status and who consumed a high carbohydrate diet (405.88 ± 57.16 g/day) during the third trimester gave birth to babies with a lower birth length. This could suggest the benefits of low carbohydrate intake in Indonesian women with >2 risk alleles of *VDR*-GRS and low vitamin D status, but would need validation from further studies [33]. 

A case–control study of 261 Indonesian adolescents showed significant interaction (P_interaction_ = 0.006) between fat intake and the *UCP2* SNP rs659366 on obesity risk. This study also indicated that carriers of *UCP2* SNP rs659366 who consumed a high-fat diet had a lower chance of becoming obese compared to non-carriers with normal fat intake [30]. *UCP2* gene variants are very commonly studied in association with obesity. The best--understood mechanisms of *UCP2*-mediated regulation of obesity include: (a) direct activation of melanocortin-4 receptor that increases energy expenditure and decreases food intake and (b) negative regulation of glucose-dependent insulin secretion in the beta cells of the pancreas and positive regulation of glucagon from the alpha cells [54]. It is also understood that *UCP2* expression has a positive correlation with weight loss [55]. 

#### 3.2.3. Singapore

Four Singaporean studies examined gene–diet interactions on obesity-related outcomes [34,35,36,37]. A study on 7817 Singaporeans examining the interaction between *CCDC* SNP rs4740619 variant and cholesterol intake on BMI in the Chinese ethnicity showed a statistically significant interaction (P_interaction_ = 0.043) between SNP rs4740619 and cholesterol intake on change in BMI. This study also indicated that this new locus identified does not commonly interact with dietary factors but proves an association in the SE Asian population [34]. An initially proposed mechanism according to the HaploReg analysis (tool to investigate the non-coding genome annotations from published GWAS (genome-wide association study) or novel variants) describes a possible alteration in the binding affinity of peroxisome proliferator-activated receptors regulating multiple metabolic pathways in obesity, but further studies are required to precisely determine the actual mechanism of *CCDC* SNP rs4740619 variant in obesity [56]. 

A 10-year prospective cohort study on 5264 individuals of Chinese ethnicity examined the interactions between *FADS* SNP rs174570 and total fish, food-sourced eicosapentaenoic acid (EPA) + docosahexaenoic acid (DHA) on BMI. The study showed significant interaction (P_interaction_ = 0.035) and a long-term increase in BMI in individuals carrying the signature ‘T’ allele with high fish/n-3 polyunsaturated fatty acids (PUFA) intake [35]. It is also well understood that *FADS1* and *FADS2* are involved in the rate-limiting steps of the fatty acid metabolic pathway and are consistently associated with plasma and tissue levels of arachidonic acid and EPA [57]. 

*APOA2* is associated with high-density lipoproteins, reverse cholesterol transport impairment, antioxidant properties, and fat distribution phenotypes that are associated with metabolic disease progression [58]. Here, a multi-ethnic cross-sectional study on 3605 Singaporeans of the Chinese, Malay, and Indian ethnicities examining saturated fatty acid (SFA) intake and *APOA2* SNP rs5082 on BMI showed no significant interaction [36]. A similar study in 4038 individuals also showed no significant interaction between PUFA/saturated fatty acids (SFA) and *PPAR-γ* SNPs rs1801282 and rs3856806 on BMI [37]. Further analysis of different *APOA2* gene variants may provide insights into gene–diet interactions specific to the SE Asian population.

### 3.3. Gene–Diet Interactions on Diabetes-Related Outcomes in the Southeast Asian Population

**Figure 3:**
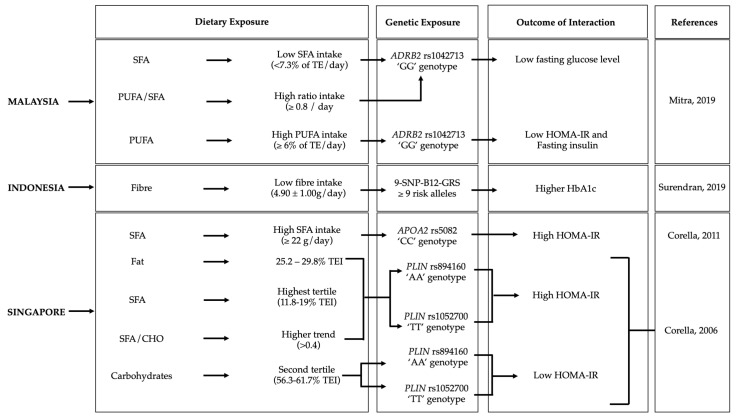
Gene–diet interactions on diabetes-related traits. The figure shows the significant gene–diet interactions (*p* < 0.05) on diabetes traits and the dietary factors that influenced the risk of diabetes in individuals carrying specific genetic variations in the Malysian [39], Indonesian [32] and Singaporean [36,41] populations. GRS, genetic risk score; BMI, body mass index; SFA, saturated fatty acids; PUFA, polyunsaturated fatty acids; HOMA-IR, homeostatic model assessment—insulin resistance; TE, total energy.

Gene–diet interactions on diabetes-related traits were observed in six Malaysian, three Indonesian, and four Singaporean studies (Figure 3, Table 2).

#### 3.3.1. Malaysia

The gene–diet interactions on diabetes-related outcomes in the Malaysian population were investigated by six studies: one RCT [26] and five cross-sectional studies [24,27,28,38,39]. 

One RCT in 128 Malaysian participants examined the interaction between *FTO* (rs9930501, rs9930506, rs9932754) and *ADRB2* (rs1042713, rs1042714) gene variants and Hipcref diet on diabetes-related outcomes in the Malay, Chinese, and Indian ethnic groups. This study showed a significant interaction (P_interaction_ = 0.048) indicating that participants had a reduction in hs-CRP level in the Hipcref-*PRS* interventional diet compared to normal diets [26]. This is a notable association because the *FTO* gene variants are not only linked with obesity but also have a strong association with diabetes [59]. When the *FTO* gene is overexpressed in INS-1 pancreatic beta cells, it upregulates transcription factor 7-like 2 (*TCF7L2*) which is a key determinant of diabetes [60]. *ADRB2* is also another gene understood to play a pivotal role in glucose homeostasis. In vivo studies have shown that pancreas-specific deletion of the *ADRB2* gene in the pancreas impacts not only glucose secretion and tolerance, but also increases *VEGF-A* production. This has a direct effect on impaired insulin production, exocytosis, and accelerates the development of diabetes-related complications like retinopathy and macular edema [61]. 

A cross-sectional study of 507 participants showed no significant interaction between *AGTR 1* SNP rs5186, *AGTR2* SNP rs1403543, and VFSD in Malay, REFD in Chinese, and VFSD in Chinese females on HbA1c levels [27]. A previous study reported several *AGTR1* gene variants expressed in several tissues such as blood vessels, kidneys, and lungs and once expressed, lead to water–sodium retention, elevated blood pressure, and microvascular disorders in diabetes [62]. Another study (n = 179) in the Chinese ethnic group examined *VEGFR2* SNP rs1870377 and meat, rice, and noodles diet on blood glucose and HbA1c also showed no significant interaction [28]. A better understanding of this relationship in the context of gene–diet interaction is important because inhibition/downregulation of the *VEGFR2* signaling axis is associated with endothelial dysfunction in diabetes [63]. 

One study conducted on 200 participants of the Chinese and Indian ethnic groups to understand the interaction of *FADS1* SNP rs174547 and LA, ALA intake on fasting blood glucose levels showed no statistically significant interactions [24]. A cross-sectional study (n = 211) performed on the Chinese ethnic group to understand the interaction of *IGF1* rs35767, *IGF1* rs7136446, *IL6* rs1800796, and DAL intake (using PRAL levels) on fasting blood glucose also showed no significant interaction [38]. *IGF1*, with structural homology to insulin, is responsible for increased peripheral glucose intake and reduction in hepatic glucose production for better insulin sensitivity. When *IGF1* levels are lower, it is often associated with higher anthropometric variables correlating with insulin resistance [64]. *IL6* is a pro-inflammatory cytokine with a known mechanism to develop insulin resistance and is involved in the pathogenesis of diabetes. This is often the result of its irregular expression (usually genetic) and long-term exposure leading to inflammation that induces insulin resistance and increases the overall risk of diabetes [65]. 

The fifth cross-sectional study (n = 126) on Malaysian, Chinese, and Indian ethnic groups to understand the interaction between *ADRB2* SNP rs1042713 and saturated fat, PUFA intake on diabetes-related outcomes showed significant gene–diet interactions on fasting blood glucose (P_interaction_ = 0.011), HOMA-IR (P_interaction_ = 0.026) and fasting insulin (P_interaction_ = 0.036). This study also showed that G allele carriers of *ADRB2* SNP rs1042713 were associated with increased odds of developing insulin resistance [39]. Understanding this gene–diet interaction in diabetes is important because *ADRB2* has shown close associations with diabetes by directly influencing anthropometric measures, fasting insulin level, and insulin resistance [61]. 

#### 3.3.2. Indonesia

Three cross-sectional studies were performed on Minangkabau women of Indonesia to understand the significance of gene–diet interaction in diabetes-related outcomes. 

One cross-sectional study (n = 117) showed significant interaction (P_interaction_ = 0.042) between vitamin B12 GRS and fiber intake on HbA1C levels. Interestingly, the study also showed that individuals with ≥9 risk alleles who consumed a low-fiber diet had higher HbA1c levels indicating a significant interaction between *B12* GRS and dietary factor [32]. Vitamin B12 deficiency in diabetic patients with metformin is quite common compared to the relationship between vitamin B12 deficiency in individuals who are not administered metformin medication. It was interesting to note that a previous study in the Chinese population (n = 16,699) demonstrated that individuals who did not take metformin as a part of their treatment regime had significantly higher B12 deficiency [66]. This poses a need for further studies on larger groups of SE Asians to understand the interaction between B12 GRS and dietary factors on diabetes-related outcomes.

The second study (n = 110) showed no significant interaction between *Metabolic GRS [FTO*, *TCF7L2*, *MC4R*, *KCNQ1*, *CDKN2A/B*] and dietary factors on diabetes-related outcomes (glucose, HbA1C, fasting insulin) [12]. The third cross-sectional study (n = 110) on Minangkabau women who were carriers of more than six risk alleles of a 15-SNP-cardiometabolic disease GRS also showed no significant interaction with protein intake on glucose levels, HbA1c levels, and fasting insulin [21]. These studies in the Indonesian population show the importance of understanding gene–diet interaction on diabetes-related outcomes, but further analysis on larger populations is required to validate these results. 

#### 3.3.3. Singapore

A total of four studies were identified: three cross-sectional studies [36,37,41], and one prospective cohort study [40]. A total of fifteen gene–diet interactions on diabetes- related traits have been identified in these studies, mostly by Corella et. al [41]. This extensive cross-sectional study on 4017 participants of the Chinese, Malay, and Indian ethnic groups showed several significant interactions between gene variants (*PLIN* rs894160, rs1052700) and dietary factors (total fat, carbohydrates, SFA) [41].

Another study (n = 3605) identified a significant interaction (P_interaction_ = 0.026) between apolipoprotein A2 (*APOA2*) SNP rs5082 and high SFA intake on homeostasis model assessment-estimated insulin resistance (HOMA-IR) in individuals who are carriers of the *CC* genotype of the variant in the Chinese, Asian, and Indian ethnicities [36]. These results are in line with another French–Caucasian case–control cohort (n = 12,387) study where the authors found an association between *APOA2* and diabetes, specifically the SNP rs5082 variant [67]. In vivo studies have also understood the mechanism of *APOA2* in diabetes where overexpression of the *APOA2* gene significantly resulted in elevated fasting blood glucose and a two-fold increase in plasma insulin levels that are key features of insulin resistance [68]. 

Peroxisome proliferator-activated receptor gamma (*PPAR-γ*) has been a prime subject of diabetes research because its ligands have been shown to be potential insulin sensitizers for the treatment of diabetes [69]. Contrastingly, Tai et. al (n = 4038), revealed that there were no interactions between the PUFA/SFA intake ratio and the *PPAR-γ* SNPs rs1801282 and rs3856806 on fasting insulin [37]. In addition, a prospective cohort study (n = 38,434) with a mean follow-up of 10.72 years examined DM-37-SNP GRS and dietary patterns (alcohol, vegetable-fruit-soy pattern, meat dim-sum pattern) on diabetes and found no significant interactions [40].

The above studies provide a complete picture of the nutrigenetic status of obesity and diabetes in the SE Asian population. Although the studies are in their infancy and are required to be understood by larger populations and all countries of the ASEAN, this research provides comparable results with similar gene–diet interactions with other parts of the world. A meta-analysis in the French population (n = 3069) confirmed an interaction (P_interaction_ = 0.0005) between low LA intake and *FADS1* rs174547 on low WC and BMI. The same study also indicated that minor allele carriers of *FADS1* SNP rs174547 benefitted from a lower dietary intake of LA [70]. A previous systematic analysis of a GLACIER study in the Swedish population (n = 5160) indicated that high PUFA intake modified the association between *FADS1*,*2*,*3* gene cluster variants (rs74771917, rs3168072, rs12577276, rs7115739, rs174602, and rs174570) and triglycerides [71]. Though not all *PLIN* variants have been associated with diabetes, some studies in American (n = 431) [72] and Chinese (n = 993) [73] women have shown a significant association between certain *PLIN* variants and the risk of diabetes. This emphasizes the need to validate the results of the above-mentioned gene–diet interaction on larger groups of the SE Asian population.

## 4. Precision Nutrition Approach for the Southeast Asian Population 

The understanding of genetic diversity between individuals and among different ethnic groups should be established before designing dietary and nutritional requirements because different individuals respond differently to lifestyle interventions. Human genome sequencing plays a pivotal role in understanding genetic variations among different ethnic groups and has paved the way for the concept of personalized nutrition to frame effective lifestyle intervention strategies [74]. Developments in omics technology provide a better understanding of the whole genome of individuals as well as different ethnic groups along with the transcriptome, proteome, metabolome, and metagenome [75]. Integration of Artificial Intelligence along with gene nutrient analysis, especially in populations such as SE Asia will be useful to develop public health strategies and personalized nutrition plans for cardiometabolic diseases such as obesity and diabetes.

A high-throughput genetic screening has been developed to understand the role of SNPs in cardiometabolic diseases. However, molecular and pathophysiological mechanisms to understand gene–nutrient interactions and its influence on cardiometabolic diseases remain unexplored. In LMIC such as in SE Asia, nutrigenetics is still in its infancy and requires an evidence-based approach before framing precision nutrition strategies for the population. Further, such studies on larger populations and ethnic groups as well as on different levels of nutrition transition, are crucial for the development of accurate and population-specific precision nutrition strategies effective to combat chronic, yet preventable diseases such as obesity and diabetes [2]. While the Western countries have shifted their approach towards nutrigenetics, developing countries like SE Asia still favor traditional methods for evaluating, categorizing, and managing obesity and diabetes. Costly gene testing, lack of knowledge, and experts in this field are the primary impediments of nutrigenetics implementation, particularly in LMIC. Even though this field is expanding globally, there are not many researchers in this discipline in SE Asia. Moving forward, the nutrigenetics approach should be considered for government health programs, particularly those aimed at noncommunicable diseases (NCDs). Currently, lifestyle diseases are a major burden to all countries, and long-term investments in accelerating nutrigenetics research and generating scientific evidence may provide a solution to obesity and its comorbidities through precision nutrition.

## 5. Limitations

**Figure 4:**
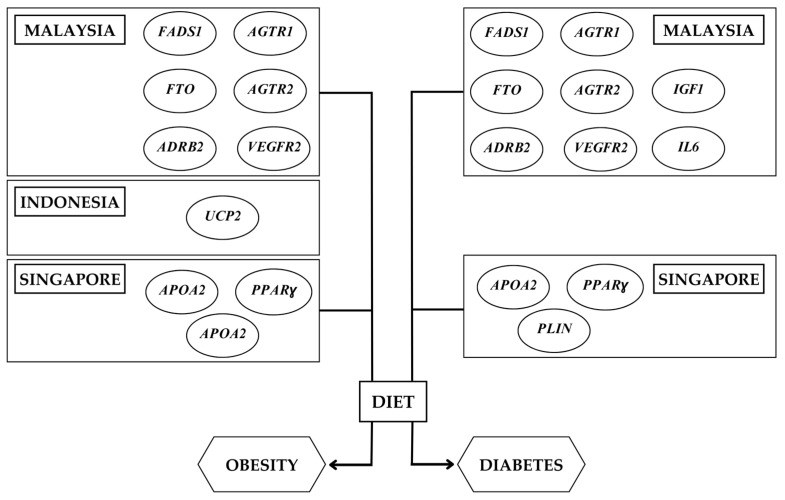
Common genes involved in gene–diet interactions associated with obesity and diabetes in the Southeast Asian population (Malaysia, Indonesia, and Singapore). *AGTR1*, angiotensin II receptor type 1; *AGTR2*, angiotensin II receptor type 2; ADRB2, adrenoceptor beta 2; *FADS1*, fatty acid desaturase 1; *FTO*, fat mass and obesity-associated gene; *VEGFR2*, vascular endothelial growth factor receptor 2; *APOA2*, apolipoprotein A2; *PPARg*, peroxisome proliferator-activated receptor-gamma.

Our study sought to analyze the gene–diet interactions on metabolic disease-related parameters in the SE Asian population. This review included twenty studies conducted in Malaysia, Indonesia, and Singapore (Figure 4) with four Indonesian studies focusing on Minangkabau women—a minority ethnic group. To the best of our knowledge, there are no articles examining gene–diet interactions on metabolic-disease-related outcomes in eight out of 11 ASEAN countries. Hence, the results of this review cannot be applied to the entire SE Asian population due to a lack of consistency and replication in dietary exposures and individual SNPs. Given these limitations, there was no possibility of a meta-analysis. Most studies performed had a cross-sectional study design and the sample size was insufficient to apply the results to the entirety of the SE Asian population. Some studies explored the relationship between SNPs and dietary factors, but there could have been an influence of other SNPs on the same outcome that remains unexplored. On performing ROBINS-1 risk assessment, one prospective cohort study was found to be at a higher risk of bias where participants were not screened for gestational diabetes which could have been a confounder in the study [33]. Future research needs to consider the general limitations highlighted in the present study and emphasize ethnic differences when looking at multi-ethnic populations.

## 6. Conclusions

This is the first systematic analysis of the effects of gene–diet interactions on obesity and diabetes in the Southeast Asian population. This review highlights several population-, sex-, and ethnicity-specific gene–diet interactions that are significant in Malaysian, Indonesian, and Singaporean populations and provide a complete picture of nutrigenetic research conducted in SE Asia. The commonly reported interactions were between macronutrients and GRS such as B12-GRS, vitamin D GRS, and a metabolic-GRS, and there were multiple interactions between *UCP2* SNP rs659366 and dietary factors on obesity traits in the Indonesian population, making the *UCP2* gene a candidate for further studies to understand the mechanisms of interaction. A deeper understanding of the *UCP2* gene–diet interaction and studies on larger groups of the SE Asian population may provide insights into personalized nutrition strategy development. Additionally, some Malaysian studies examined gene–diet interactions with specific dietary patterns in population subgroups including *FTO*, *ADRB2*, and Hipcref diet, *AGTR1*, *AGTR2* genes, and VFSD, REFD diets, and *VEGFR2* with a meat, rice, noodles diet to better understand its influence on obesity and diabetes [26,27,28]. Similar such studies are crucial in larger populations and ethnic groups for the development of accurate, population-specific precision nutrition strategies to effectively combat chronic, yet preventable diseases such as obesity and diabetes.

## Figures and Tables

**Table 1:** Summary table of gene–diet interactions on obesity in populations from Southeast Asians by country.

Gene	Genetic Variation	Study Design	n (Men/Women)	Ethnicity	Age (Years)	Dietary Factors	Outcomes	P_interaction_	Interpretation	Ref.
**Malaysia**										
*FADS1*	rs174547	C-S	200 (69/131)	Chinese Indians	>18	Linoleic acid (7.9 ± 3.6 g/day)	WC	0.177	No significant interaction; vegetarians with TT genotype had higher odds of MetS, larger WC, and low HDL-c	[24]
						α-Linolenic Aaid (0.4–0.8 g/day)		0.258		
*Maternal VDR*	rs2228570	C-S	217 (107/110)	Malay Chinese Indians Kadazan Bajau Suluk Mixed Ethnic	28.9 ± 4.2	Maternal 25OHD (< or >30 nmol/L), infant 25OHD (< or >30 nmol/L)	Infant birth weight	**0.018**	Significant interaction. Inverse association of maternal Vit D deficiency with neonatal birth anthropometry; neonatal G allele carriers associated with higher birth weight	[25]
*Maternal GC*	rs7041									
*Maternal GC*	rs4588									
*Cord VDR*	rs2228570									
*Cord GC*	rs7041									
*Cord GC*	rs4588									
*FTO*	rs9930501	RCT	103 (16/87)	Malay Chinese Indians	>18	Hipcref (high-protein calorie-restricted) diet	BMI	0.125	No significant interactions	[26]
	rs9930506						Body weight	0.058		
	rs9932754						WC	0.224		
*ADRB2*	rs1042713						WHR	0.369		
							Fat mass	0.234		
	rs1042714						BFP	0.468		
							Muscle mass	0.068		
*AGTR1*	rs5186	C-S	507 (154/353)	Malay Chinese Indian	30–65	Vegetables, fruits, soy diet (VFSD) in Malay	BMI	0.994	No significant interactions; Malay and Chinese showed high risk for lipids with gene–diet interactions but not Indians	[27]
						Rice, egg and fish diet (REFD) in Chinese		0.66		
*AGTR2*	rs1403543					VFSD in Chinese females		0.053		
*VEGFR2*	Rs1870377	P-C	179	Chinese	30–65	Meat, rice, and noodles diet	BMI	0.408	No significant interactions	[28]
**Indonesia**										
*UCP2*	rs659366 (−866G/A) AA + GA Genotype	P-C	203	Indonesian	20–56	Total energy intake (kcal) [2570 ± 1066 (baseline) to 2120 ± 1042 (after 2 years)]	Changes in body weight (kg)	0.263	No significant interactions	[29]
							Changes in % body fat	0.303		
							Changes in WHR	0.464		
						Fat intake % [23.9 ± 11.2]	Changes in body weight (kg)	0.896		
							Changes in % body fat	0.965		
							Changes in WHR	0.996		
						Carbohydrate intake % [63.9 ± 11.2 (baseline) to 64.2 ± 10.4 (after 2 years)]	Changes in body weight (kg)	0.433		
							Changes in % body fat	0.839		
							Changes in WHR	0.665		
						Protein intake % [12·3 ± 3.4 (baseline) to 12.4 ± 3.7 (after 2 years)]	Changes in body weight (kg)	0.076		
							Changes in % body fat	0.360		
							Changes in WHR	0.355		
						Physical activity (MET-min/week)	Changes in body weight (kg)	0.251		
							Changes in % body fat	0.979		
							Changes in WHR	0.684		
*UCP2*	rs659366 (−866G/A) GG Genotype	P-C	120	Indonesian	20–56	Total energy intake (kcal) [2570 ± 1066 (baseline) to 2120 ±1042 (after 2 years)]	Changes in body weight (kg)	**0.016**	Significant interaction between *UCP2* gene variation and total energy intake on body weight change and BFP Significant interaction between *UCP2* gene variation and physical activity on WHR	
							Changes in % body fat	**0.034**		
							Changes in WHR	0.070		
						Fat intake % [23.9 ±11.2]	Changes in body weight (kg)	0.682		
							Changes in % body fat	0.974		
							Changes in WHR	0.753		
						Carbohydrate intake % [63.9 ± 11.2 (baseline) to 64.2 ±10.4 (after 2 years)]	Changes in body weight (kg)	0.580		
							Changes in % body fat	0.771		
							Changes in WHR	0.826		
						Protein intake % [12·3 ± 3.4 (baseline) to 12.4 ± 3.7 (after 2 years)]	Changes in body weight (kg)	0.830		
							Changes in % body fat	0.913		
							Changes of WHR	0.103		
						Physical activity (MET-min/week)	Changes in body weight (kg)	0.666		
							Changes in % body fat	0.653		
							Changes in WHR	**0.040**		
*GRS*	Vitamin D GRS [DHCR7, CYP2R1, CYP24A1, GC, CASR]	C-S	110	Minangkabau women	25–60	Carbohydrate [(235.2 g ± 73.5)]	BFP	**0.049**	A significant interaction between Vitamin D *GRS* and carbohydrate intake on log_BFP_; carriers of more than 2 risk alleles and consumed high carbohydrate amounts had significantly high log_BFP_	[12]
	Metabolic GRS [FTO, TCF7L2, MC4R, KCNQ1, CDKN2A/B]					Carbohydrate [(235.2 g ± 73.5)] and protein [77.87 g ± 220.5]	BMI WC BFP	0.997	No significant interactions	
*UCP2*	rs659366 (−866G/A)	C-C	261 (145/116)	Indonesian	15–21	Fat intake % [(>24.14 ± 5.95]	Obesity	**0.006**	A significant interaction between *UCP2* rs659366 (−866G/A) and fat intake on obesity	[30]
*UCP2*	rs659366 (−866 AA + GA)	C-S	455 (223/232)	Indonesian	19–56	Coffee intake ml (34.5 ± 89.6)	BMI	0.214	Significant interaction between *UCP2* rs659366 (−866 AA + GA) and coffee intake on body fat	[31]
							Body fat	**0.015**		
							WC	0.302		
							Hip circumference	0.253		
	rs659366 (−866 GG)					Coffee intake ml (37.1 ± 106.4)	BMI	0.231		
							Body fat	0.313		
							WC	0.510		
							Hip circumference	0.421		
*GRS*	9-SNP-B12-GRS	C-S	117	Minangkabau women	25–60	Fat (59.00 g ± 33.10)	BMI	0.933	A significant interaction between *B12 GRS* and protein energy (%) on BFP	[32]
							WC	0.444		
							BFP	0.275		
						Carbohydrate (233 g ± 71)	BMI	0.685		
							WC	0.875		
							BFP	0.064		
						Protein energy (76.90 g ± 36.50)	BMI	0.993		
							WC	0.395		
							BFP	**0.034**		
						Fiber energy (8.80 g ± 4.50)	BMI	0.155		
							WC	0.547		
							BFP	0.697		
	9-SNP-Metabolic-GRS					Fat (59.00 g ± 33.10)	BMI	0.422	A significant interaction between metabolic *GRS* and protein energy (%) on WC	
							WC	0.812		
							BFP	0.775		
						Carbohydrate ((233 g ± 71)	BMI	0.230		
							WC	0.072		
							BFP	0.844		
						Protein energy (76.90 g ± 36.50)	BMI	0.110		
							WC	**0.032**		
							BFP	0.568		
						Fiber energy (8.80 g ± 4.50)	BMI	0.273		
							WC	0.648		
							BFP	0.423		
*GRS*	6SNP-Vitamin D-GRS (≤3)	P-C	183	Minangkabau women	29.6 ± 5.56	Maternal carbohydrate intake during third trimester	Infant birth weight	0.611	Significant interactions between *VDR GRS* and carbohydrate intake on new-born birth length Pregnant women with a high genetic risk of vitamin D deficiency with high carbohydrate intake gave birth to babies with lower birth lengths	[33]
							Infant birth length	0.065		
	6SNP-Vitamin D-GRS (≥4)						Infant birth weight	0.872		
							Infant birth length	0.073		
	4-SNP-GRS[DHCR7, GC, CYP24A1 and CYP2R1] (<3)						Infant birth weight	0.841		
							Infant birth length	0.256		
	4-SNP-GRS[DHCR7, GC, CYP24A1 and CYP2R1] (≥3)						Infant birth weight	0.795		
							Infant birth length	0.079		
	2-SNP-VDR-GRS (<2)						Infant birth weight	0.810		
							Infant birth length	**0.032**		
	2-SNP-VDR-GRS (≥2)						Infant birth weight	0.775		
							Infant birth length	0.099		
*GRS*	15-SNP-cardiometabolic disease related traits-GRS	C-S	110	Minangkabau women	25–60	Carbohydrate (53.97 ± 9.44)	BMI	0.961	Significant interaction between *GRS* and protein intake on obesity-related outcome	[21]
							WC	0.224		
						Protein (13.51 ± 1.18% TEI)	BMI	0.282		
							WC	**0.002**		
						Fat (28.95 ± 7.99)	BMI	0.721		
							WC	0.577		
							BMI	0.876		
						Fiber g (8.78 ± 4.29)	WC	0.614		
**Singapore**										
*CCDC171*	rs4740619	C-S P-C	7817	Chinese	24–95	Cholesterol (lowest consumption group 184.35 ± 116.00 to highest consumption group 260.61 ± 150.56)	BMI	**0.043**	Significant interaction was observed; *CCDC171* rs4740619 interaction with cholesterol showed increased BMI level in subjects	[34]
*FADS*	rs174570	P-C	5264	Chinese	30–55	Total fish (0.16 servings per day ±0.07) Food sourced EPA + DHA (0.33 g/d ±0.20)	BMI	**0.035**	Significant interaction was observed.; long-term BMI changes in people with high fish/n-3 PUFA intake carrying signature allele show increased weight gain and risk of obesity	[35]
*APOA2*	rs5082(−265T > C)	C-S	3605 (1714/1891)	Chinese Malay Indian	18–69	SFA intake (22 g)	BMI	0.758	No significant interactions	[36]
*PPAR-* *ϒ*	rs1801282(Pro12Ala)	C-S	4038 (1869/2169)	Chinese Malay Indian	18–69	PUFA/SFA	BMI	0.873	No significant interactions	[37]
	rs3856806(C1431T)							0.472		

Notes: Hipcref (high-protein calorie-restricted) Diet: energy deficit of 300–500 kcal/day, 30% energy from protein, 30% energy from fat, 40% energy from carbohydrate, vitamin E ≥ 15 mg/day, and fiber ≥ 25 g/day. Control diet: dietary advice on weight loss based on the Malaysian Dietary Guidelines 2010 (<1500 kcal/day with a macronutrient composition of approximately 10–15% energy from protein, 20–30% energy from fat, and 55–70% energy from carbohydrate) c PRS (polygenic risk score): *FTO* rs9930501, rs9930506, rs9932754 *ADRB2* rs1042713, rs1042714. PRAL: potential renal acid load = 0.49 protein (g/day) + 0.037 phosphorus (mg/day)—0.021 potassium (mg/day)—0.026 magnesium (mg/day)—0.013 calcium (mg/day). C-S, cross-sectional; P-C, prospective-cohort study; RCT, randomized control trial. Statistically significant gene-diet interactions are highlighted in bold.

**Table 2:** Summary table of gene–diet interactions on diabetes in Southeast Asians by country.

Genes	Genetic Variations	Study Design	n (Male/Female)	Ethnicity	Age (Years)	Dietary Factors	Outcomes	P_interaction_	Interpretation	Ref.
**Malaysia**										
*FTO*	rs9930501	RCT	103 (16/87)	Malay Chinese Indians	>18	Hipcref ^a^ (high-protein calorie-restricted) control diet ^b^	Fasting glucose	0.381	Significant interaction observed;participants showed a greater reduction in hsCRP levels with the Hipcref diet compared to normal diet	[26]
	rs9930506						Fasting insulin	0.121		
	rs9932754									
*ADRB2*	rs1042713						HOMA-IR	0.122		
	rs1042714						hs-CRP	**0.048**		
*AGTR1*	rs5186	C-S	507 (154/353)	Malay Chinese Indian	30–65	Vegetables, fruits, and soy diet (VFSD) in Malays	HbA1C	0.537	No significant interactions	[27]
						Rice, egg, and fish diet (REFD) in Chinese		0.844		
*AGTR2*	rs1403543					VFSD in Chinese females		0.989		
*VEGFR2*	rs1870377	C-S	179	Chinese	30–65	Meat, rice, and noodles diet	Blood glucose	*p* > 0.05	No significant interactions	[28]
							HbA1c	*p* > 0.05		
*FADS1*	rs174547	C-S	200 (69/131)	Chinese Indians	>18	Linoleic acid (7.9 ± 3.6 g/day)	Log _FBG_	0.807	No significant interactions	[24]
						α-Linolenic acid (0.4–0.8 g/day)		0.293		
*IGF1*	rs35767	C-S	211	Chinese	66.7 ± 6	DAL (using PRAL)	FBG	NS	No significant interactions; study shows association between DAL and high FBG, indicating a potential risk factor for diabetes	[38]
*IGF1*	rs7136446									
*IL6*	rs1800796									
*ADRB2*	rs1042713	C-S	126	Malaysian Chinese Indians	18–74	Saturated fat intake (<7.3% of total energy/day) PUFA intake (≥0.8/day) PUFA:SFA ratio (≥6% of TE/ day)	FBG	**0.011**	Significant gene diet interactions; G allele carriers of *ADRB2* rs1042713 are associated with increased odds of insulin resistance	[39]
							HOMA-IR	**0.026**		
							Fasting insulin	**0.036**		
**Indonesia**										
*GRS*	9-SNP-B12-GRS	C-S	117	Minangkabau women	25–60	Fat (59.00 ± 33.10)	log _HbA1c_	0.175	Significant interaction between B12 GRS and fiber intake on HbA1c levels; individuals with ≥9 risk alleles who consumed low fiber diet had significantly higher HbA1c levels	[32]
							log _FBG_	0.374		
							log _fasting serum insulin_	0.757		
						Carbohydrate (233 ± 71)	log _HbA1c_	0.091		
							log _FBG_	0.260		
							log _fasting serum insulin_	0.341		
						Protein (76.90 ± 36.50)	log _HbA1c_	0.150		
							log _FBG_	0.368		
							log _fasting serum insulin_	0.073		
						Fiber intake (8.80 ± 4.50)	log _HbA1c_	**0.042**		
							log _FBG_	0.380		
							log _fasting serum insulin_	0.215		
	9-SNP-Metabolic-GRS					Fat (59.00 ± 33.10)	log _HbA1c_	0.298		
							log _FBG_	0.634		
							log _fasting serum insulin_	0.108		
						Carbohydrate (233 ± 71)	log _HbA1c_	0.166		
							log _FBG_	0.771		
							log _fasting serum insulin_	0.104		
						Protein (76.90 ± 36.50)	log _HbA1c_	0.155		
							log _FBG_	0.929		
							log _fasting serum insulin_	0.890		
						Fiber intake (g/d) (4.90 ± 1.00 g/day)	log _HbA1c_	0.851		
							log _FBG_	0.215		
							log _fasting serum insulin_	0.947		
*GRS*	Metabolic GRS [FTO, TCF7L2, MC4R, KCNQ1, CDKN2A/B]	C-S	110	Minangkabau women	25–60	Carbohydrate (g/d) (233.7 ± 75.1 g)	Glucose	0.360	No significant interactions	[12]
							HbA1c	0.780		
							Fasting insulin	0.630		
						Protein (g/d) (77.2 ± 41.7 g)	Glucose	0.560		
							HbA1c	0.680		
							Fasting insulin	0.220		
						Fat (g/d) (61.2 ± 36.1 g)	Glucose	0.700		
							HbA1c	0.780		
							Fasting insulin	0.440		
						Fiber (g/d) (8.6 ± 4.3 g)	Glucose	0.830		
							HbA1c	0.530		
							Fasting insulin	0.440		
*GRS*	15-SNP-cardiometabolic disease related traits-GRS	C-S	110	Minangkabau women	25–60	Carbohydrates % (53.97 ± 9.44)	log_Glucose_	0.882	No significant interactions	[21]
							log_Insulin_	0.336		
							log_HbA1c_	0.766		
						Protein % (16.93 ± 3.32)	log_Glucose_	0.751		
							log_Insulin_	0.341		
							log_HbA1c_	0.638		
						Fat % (28.95 ± 7.99)	log_Glucose_	0.732		
							log_Insulin_	0.480		
							log_HbA1c_	0.935		
						Fiber g (8.78 ± 4.29)	log_Glucose_	0.833		
							log_Insulin_	0.216		
							log_HbA1c_	0.162		
**Singapore**										
*GRS*	DM 37-SNP GRS	P-C	38,434	Chinese	30–79	Alcohol (men: 10–25 g/d; women: 5–15 g/d)	Diabetes risk	NS	No significant interaction; but a healthy lifestyle and any genetic risk category was associated with a significantly lower risk of diabetes	[40]
						VFSD pattern				
						Meat—dim sum pattern				
*PLIN*	rs894160(11482G > A)	C-S	4107	Chinese Malay Indian	18–69	Total fat % (25.2–29.8 TEI)	Fasting glucose	0.425	Significant interaction between *PLIN* variants and dietary factors on diabetes related outcomes	[41]
							Fasting insulin	**0.01**		
							HOMA-IR	**0.007**		
						SFA % (9.4–11.8)	Fasting glucose	**0.004**		
							Fasting insulin	**0.004**		
							HOMA-IR	**0.003**		
						Carbohydrates % (56.3–61.7)	Fasting glucose	0.145		
							Fasting insulin	**0.007**		
							HOMA-IR	**0.004**		
	rs1052700(14995A > T)					Total fat % (25.2–29.8 TEI)	Fasting glucose	0.448		
							Fasting insulin	**0.014**		
							HOMA-IR	**0.012**		
						SFA % (9.4–11.8)	Fasting glucose	**0.009**		
							Fasting insulin	**0.014**		
							HOMA-IR	**0.005**		
						Carbohydrates % (56.3–61.7)	Fasting glucose	0.293		
							Fasting insulin	**0.008**		
							HOMA-IR	**0.012**		
*PPAR-* *ϒ*	rs1801282(Pro12Ala)	C-S	4038 1869/2169)	Chinese Malay Indian	18–69	PUFA/SFA	Insulin	0.089	No significant interactions	[37]
	rs3856806(C1431T)							0.175		
*APOA2*	rs5082(−265T > C)	C-S	3605 (1714/1891)	Chinese Malay Indian	18–69	SFA intake (22 g)	HOMA-IR	**0.026**	Significant interaction between *APOA2* rs5082(−265T > C) and SFA intake on HOMA-IR	[36]

Notes. ^a^ Hipcref (high-protein calorie-restricted) Diet: energy deficit of 300–500 kcal/day, 30% energy from protein, 30% energy from fat, 40% energy from carbohydrate, vitamin E ≥15 mg/day, and fiber ≥25 g/day. ^b^ control diet: dietary advice on weight loss based on the Malaysian Dietary Guidelines 2010 (<1500 kcal/ day with a macronutrient composition of approximately 10–15% energy from protein, 20–30% energy from fat, and 55–70% energy from carbohydrate) ^c^ PRS: *FTO* rs9930501, rs9930506, rs9932754 *ADRB2* rs1042713, rs1042714. PRAL: potential renal acid load = 0.49 protein (g/day) + 0.037 phosphorus (mg/day)—0.021 potassium (mg/day)—0.026 magnesium (mg/day)—0.013 calcium (mg/day). C-S, cross-sectional; P-C, prospective-cohort study; RCT, randomized control trial. Statistically significant gene-diet interactions are highlighted in bold.

## Data Availability

The original contributions presented in this study are included in the Supplementary material. Further inquiries can be directed to the corresponding author.

## Supplementary Materials

The following supporting information can be downloaded at: https://www.mdpi.com/article/10.3390/nu15132948/s1, Table S1. Number of Hits and Search Strings per Database. Table S2. Summary Outcome of Assessment with the Appraisal Tool for Cross-Sectional Studies (AXIS). Table S3. Assessment with the Comments Appraisal Tool for Cross-Sectional Studies. Table S4. Assessment using the Risk of Bias in Non-Randomized Studies—of Interventions (ROBINS-I) [29,33,34,35,40]. Table S5. Assessment using RoB 2: A revised Cochrane risk-of-bias tool for randomized trials. Section S1—Risk of bias assessment.
