# Supplementary material for: Gene–Diet Interactions on Metabolic Disease-Related Outcomes in Southeast Asian Populations: A Systematic Review
Source: Nutrients. 2023 Jun 29;15(13):2948. doi: 10.3390/nu15132948 (PMC10346616; doi:10.3390/nu15132948)
Supplement: Supplementary file 1 [file nutrients-15-02948-s001.zip › nutrients-2452634-supplementary.pdf]

Supplementary Section

Table S1. Number of Hits and Search Strings per Database

Search engine/ database	Search string	No of HITS
PubMed	((((polymorphism OR gene OR SNP OR single nucleotide polymorphism OR genetic variation OR genetic variant OR GRS OR genetic risk score OR PRS OR polygenic risk score) AND ("gene-diet interaction" OR "diet-gene interaction" OR SNP-diet interaction OR diet-SNP interaction OR "gene-nutrient interaction" OR "nutrient-gene interaction" OR "gene-lifestyle interaction" OR "gene-environment interaction")) AND (carbohydrate OR glucose OR protein OR fat OR fibre OR sugar OR SFA OR saturated fat OR monounsaturated fat OR polyunsaturated fat OR MUFA OR PUFA OR B12 OR vitamin D OR amino acids OR polyphenols OR egg intake OR caffeine intake OR green tea OR alcohol intake OR meat intake OR energy intake OR food OR diet)) AND (diabetes OR fasting glucose OR insulin OR HbA1c OR metabolic syndrome OR metabolic disease* OR glycaemic traits OR glycaemia* postprandial)) AND (Southeast Asia OR Malay* OR Brunei* OR Burm* OR cambodia* OR timor* OR indonesia* OR Laos OR Filipin* OR philippine* OR singapore* OR thai* OR vietnam*)	1,398
	((((polymorphism OR gene OR SNP OR single nucleotide polymorphism OR genetic variation OR genetic variant OR GRS OR genetic risk score OR PRS OR polygenic risk score) AND ("gene-diet interaction" OR "diet-gene interaction" OR SNP-diet interaction OR diet-SNP interaction OR "gene-nutrient interaction" OR "nutrient-gene interaction" OR "gene-lifestyle interaction" OR "gene-environment interaction")) AND (carbohydrate OR protein OR fat OR fibre OR sugar OR SFA OR saturated fat OR monounsaturated fat OR polyunsaturated fat OR MUFA OR PUFA OR diet OR B12 OR vitamin D OR amino acids OR polyphenols OR egg intake OR caffeine intake OR green tea OR alcohol intake OR meat intake OR energy intake OR food)) AND (Obesity OR weight OR BMI OR waist circumference OR waist hip ratio OR hip circumference OR adiposity OR metabolic diseases OR body fat OR body composition)) AND (Southeast Asia OR Malay* OR Brunei* OR Burm* OR cambodia* OR timor* OR indonesia* OR Laos OR Filipin* OR philippine* OR singapore* OR thai* OR vietnam*)	510
Google Scholar	gene-diet interaction diabetes Southeast Asia OR Malay* OR Brunei* OR Burm* OR cambodia* OR timor* OR indonesia* OR Laos OR Filipin* OR philippine* OR singapore* OR thai* OR vietnam*	537
	gene-diet interaction BMI Southeast Asia OR Malay* OR Brunei* OR Burm* OR cambodia* OR timor* OR indonesia* OR Laos OR Filipin* OR philippine* OR singapore* OR thai* OR vietnam*	570
PubMed	"Gene - nutrient interaction Malaysia"<br>"Gene-nutrient interactions Singapore"<br>"Gene-nutrient interactions Indonesia"<br>"Gene- nutrient interactions Thailand"<br>"Gene- nutrient interactions Vietnam"<br>"Gene-nutrient interactions Philippines"<br>"Gene-nutrient interactions Laos"	516

	"Gene-nutrient interactions Timor Leste"<br>"Gene-nutrient interactions Cambodia"<br>"Gene - diet interaction Malaysia"<br>" Gene - diet interaction Singapore"<br>" Gene - diet interaction Indonesia"<br>" Gene - diet interaction Thailand"<br>" Gene - diet interaction Vietnam"<br>" Gene - diet interaction Philippines"<br>" Gene - diet interaction Laos"<br>" Gene - diet interaction Timor Leste"<br>" Gene - diet interaction Cambodia"	
Google Scholar	"Gene - nutrient interaction Malaysia"<br>"Gene-nutrient interactions Singapore"<br>"Gene-nutrient interactions Indonesia"<br>"Gene- nutrient interactions Thailand"<br>"Gene- nutrient interactions Vietnam"<br>"Gene-nutrient interactions Philippines"<br>"Gene-nutrient interactions Laos"<br>"Gene-nutrient interactions Timor Leste"<br>"Gene-nutrient interactions Cambodia"<br>"Gene - diet interaction Malaysia"<br>" Gene - diet interaction Singapore"<br>" Gene - diet interaction Indonesia"<br>" Gene - diet interaction Thailand"<br>" Gene - diet interaction Vietnam"<br>" Gene - diet interaction Philippines"<br>" Gene - diet interaction Laos"<br>" Gene - diet interaction Timor Leste"<br>" Gene - diet interaction Cambodia"	15,500
TOTAL		19,031

Section 1 – Risk of bias assessment

Appraisal tool for Cross-sectional studies (AXIS)

Introduction

1. Were the aims/ Objectives of the study clear?

Methods

2. Was the study design appropriate for the stated aim(s)?
3. Was the sample size justified?
4. Was the target/reference population clearly defined? (Is it clear who the research was about?
5. Was the sample frame taken from an appropriate population base so that it closely represented the target/reference population under investigation?
6. Was the selection process likely to select subjects/participants that were representative of the target/reference population under investigation?
7. Were measures undertaken to address and categorize non-responders?
8. Were the risk factor and outcome variables measured appropriate to the aims of the study?
9. Were the risk factor and outcome variables measured correctly using instruments/ measurements that had been trialled, piloted or published previously? (Only dietary, nutritional, physical activity assessment were evaluated)
10. Is it clear what was used to determined statistical significance and/or precision estimates? (e.g., p-values, CIs)
11. Were the methods (including statistical methods) sufficiently described to enable them to be repeated?

Results

12. Were the basic data adequately described?
13. Does the response rate raise concerns about non-response bias?
14. If appropriate, was information about non-responders described?
15. Were the results internally consistent?
16. Were the results for the analyses described in the methods, presented?

Discussion

17. Was the author´s discussion and conclusions justified by the results?
18. Were the limitations of the study discussed?
19. Were there any funding sources or conflicts that may affect the authors interpretations of the results?
20. Was ethical approval or consent of participants attained?

Table S2. Summary Outcome of Assessment with the Appraisal Tool for Cross-Sectional Studies (AXIS)

	Yap et al 2017	Lim et al 2021	Lee et al 2022	Ching et al 2019	Alathari et al 2021	Surendran et al 2019	Mitra et al 2019	Alsulami et al 2020	Huriyati et al 2016	Corella et al 2011	Corella et al 2006	Muhammad et al 2019	Tai et al 2004	Yap et al 2011
1)	Y	Y	Y	Y	Y	Y	Y	Y	Y	Y	Y	Y	Y	Y
2)	Y	Y	Y	Y	Y	Y	Y	Y	Y	Y	Y	Y	Y	Y
3)	Y	Y	Y	Y	Y	Y	Y	Y	Y	Y	Y	Y	Y	Y
4)	Y	Y	Y	Y	Y	Y	Y	Y	Y	Y	Y	Y	Y	Y
5)	Y	Y	Y	Y	Y	Y	Y	Y	Y	Y	Y	Y	Y	Y
6)	Y	Y	Y	Y	Y	Y	Y	Y	Y	Y	Y	Y	Y	Y
7)	Y	Y	Y	Y	Y	Y	Y	Y	Y	Y	Y	Y	Y	Y
8)	Y	Y	Y	Y	Y	Y	Y	Y	Y	Y	Y	Y	Y	Y
9)	Y	Y	Y	Y	Y	Y	Y	Y	Y	Y	Y	Y	Y	Y
10)	Y	Y	Y	Y	Y	Y	Y	Y	Y	Y	Y	Y	Y	Y
11)	Y	Y	Y	Y	Y	Y	Y	Y	Y	Y	Y	Y	Y	Y
12)	Y	Y	Y	Y	Y	Y	Y	Y	Y	Y	Y	Y	Y	Y
13)	N	N	N	Y	N	N	N	N	N	N	N	N	N	N
14)	Y	Y	Y	Y	Y	Y	Y	Y	Y	Y	Y	Y	Y	Y
15)	Y	Y	Y	Y	Y	Y	Y	Y	Y	Y	Y	Y	Y	Y
16)	Y	Y	Y	Y	Y	Y	Y	Y	Y	Y	Y	Y	Y	Y
17)	Y	Y	Y	Y	Y	Y	Y	Y	Y	Y	Y	Y	Y	Y
18)	Y	Y	Y	Y	Y	Y	Y	Y	N	Y	Y	Y	Y	Y
19)	N	N	N	N	N	N	N	N	N	N	N	N	N	N
20)	Y	Y	Y	Y	Y	Y	Y	Y	Y	Y	Y	Y	Y	Y

Note: Numbered questions are listed apart.

Table S3. Assessment with the Comments Appraisal Tool for Cross-Sectional Studies

Study	**Introduction**	**Method**										**Results**					**Discussion**			
	1.	2.	3	4.	5.	6.	7.	8.	9.	10.	11	12.	13	14.	15.	16.	17.	18.	19.	20.
Ching et al 2019	Y	Y	Y	Y	Y	Y	Y	Y	Y	Y	Y	Y	**Y**	Y	Y	Y	Y	Y	N	Y
Description	33% from the initial sample size was excluded. 32 out of 273 respondents had misreported their energy intake. 41 respondents had incomplete three-day dietary recall data.																			
Huriyati et al 2020	Y	Y	Y	Y	Y	Y	Y	Y	Y	Y	Y	Y	N	Y	Y	Y	Y	N	N	Y
Description	Did not discuss own limitations.																			

Note: Questions are listed above, Table S2 summarises Table S3.

Table S4. Assessment using the Risk of Bias in Non-Randomized Studies – of Interventions (ROBINS-I)

ROBINS-I assessment	**1.1 Is there potential for confounding of the effect of exposure in this study? If N or PN to 1.1: the study can be considered to be at low risk of bias due to confounding and no further signalling questions need be considered Y / PY / PN / N**
**Muhammad et al 2021**	PN
[Description]	"All anthropometric measurements were conducted by trained personnel using calibrated instruments" "…data was collected by a face-to-face interview between trained nutritionists and subjects. Those questionnaires were developed, validated and used in previous studies."
**Aji et al 2022**	PY
[Description]	The study participants were not screened for Gestational diabetes mellitus and hence this could be a confounder in the study.
**Chang et al 2018**	PN
[Description]	"Conducted various sensitivity analyses and subgroup analyses. Used a DXA and measured weight to the nearest 0.1kg.
**Huang et al 2019**	PN
[Description]	"Potential confounders considered in multivariable models were age, baseline physical activity, baseline television watching, baseline smoking, baseline alcohol intake, baseline alternate healthy eating index and baseline total energy intake, sugar sweetened beverages (if available), fried food intake (if available). We further tested the genetic associations … using multiple linear regression model after adjustment of potential confounders" Statistical analysis was adjusted for a number of possible confounding variables. "We have carefully adjusted for multiple dietary and lifestyle factors"
**Li et al 2020**	PN
[Description]	"We controlled for potential confounding factors and sought to minimize the reverse causation bias by excluding participants with major chronic diseases at baseline, which might lead to lifestyle changes" "detailed collection of dietary data through face-to-face interviews used an FFQ that was specifically developed and validated in 2 cohorts. The anthropometric information was measured by trained staff…"

Continuation of Table S4.

Study: Aji et al		
		[Description]
**Bias due to confounding**		
1.1 Is there potential for confounding of the effect of exposure in this study? If N or PN to 1.1: the study can be considered to be at low risk of bias due to confounding and no further signalling questions need be considered Y / PY / PN / N	PY	The study participants were not screened for Gestational diabetes mellitus and hence this could be a confounder in the study.
If Y/PY to 1.1, answer 2.1 and 1.3 to determine whether there is a need to assess time-varying confounding:		
1.2. If Y or PY to 1.1: Was the analysis based on splitting, follow up time according to exposure received?	N	
If N or PN to 1.2, answer questions 1.4 to 1.6, which relate to baseline confounding		
1.3. If Y or PY to 1.2: Were exposure discontinuations or switches likely to be related to factors that are prognostic for the outcome?	Y	"Third-trimester dietary intake status" via Food frequency questionnaire
If N or PN to 1.3, answer questions 1.4 to 1.6, which relate to baseline confounding		
**Questions relating to baseline confounding only**		
1.4. Did the authors use an appropriate analysis method that controlled for all the important confounding domains?	Y	"Tested using linear regression after adjusting for potential confounding factors such as age, pre pregnancy BMI, total energy intake, vitamin D, GA at birth and gender of the infant"
1.5. If Y/PY to 1.4: Were confounding domains that were controlled for measured validly and reliably by the variables available in this study?	Y	
1.6. Did the authors control for any post-intervention variables that could have been affected by the intervention?	N	They did not control for gestational diabetes mellitus.
**Bias in selection of participants into the study**		
2.1. Was selection of participants into the study (or into the analysis) based on variables measured after the start of the exposure?	Y	
If N or PN to 2.1 go to 2.4		

Table S5. Assessment using RoB 2: A revised Cochrane risk-of-bias tool for randomized trials

Study details

Reference

Tan, P.Y.; Mitra, S.R. The Combined Effect of Polygenic Risk from FTO and ADRB2 Gene Variants, Odds of Obesity, and Post-Hipcref Diet Differences. *Lifestyle genomics* **2020**, *13*, 84–98, doi:10.1159/000505662.

Study design

X Individually-randomized parallel-group trial

☐ Cluster-randomized parallel-group trial

☐ Individually randomized cross-over (or other matched) trial

For the purposes of this assessment, the interventions being compared are defined as

Experimental: ☐ Comparator: X

Specify which outcome is being assessed for risk of bias

"Effect of the interaction between polygenic risk score and dietary group on the post-intervention differences in dietary parameters in response to the 6-month Hipcref diet or the control diet."

Specify the numerical result being assessed

A General linear regression model (Table 7). p interaction (PRS × intervention group)

Is the review team's aim for this result…?

X to assess the effect of *adhering to intervention* (the 'per-protocol' effect)

If the aim is to assess the effect of *adhering to intervention*, select the deviations from intended intervention that should be addressed (at least one must be checked):

X occurrence of non-protocol interventions

X failures in implementing the intervention that could have affected the outcome

X non-adherence to their assigned intervention by trial participants

Which of the following sources were <u>obtained</u> to help inform the risk-of-bias assessment? (tick as many as apply)

X Journal article(s) with results of the trial

X Trial protocol

X Statistical analysis plan (SAP)

Risk of bias assessment

Responses underlined are potential markers for low risk of bias, and responses in bold are potential markers for a risk of bias. Where questions relate only to sign posts to other questions, no formatting is used.

Domain 1: Risk of bias arising from the randomization process

Signalling questions	Comments	Response options
1.1 Was the allocation sequence random?	"recruited at random through advertisements and flyers distributed at the University of …, supermarkets, and schools in the vicinity"	Y
1.2 Was the allocation sequence concealed until participants were enrolled and assigned to interventions?	"…covariate adaptive randomization technique" "All participants were blinded to the allocation of the dietary arm of the study"	Y
1.3 Did baseline differences between intervention groups suggest a problem with the randomization process?	PN, no imbalances are apparent	PN
Risk-of-bias judgement	Low risk	Low

Domain 2: Risk of bias due to deviations from the intended interventions (*effect of assignment to intervention*)

Signalling questions	Comments	Response options
2.1. Were participants aware of their assigned intervention during the trial?	N, "All participants were blinded to the allocation of the dietary arm of the study"	N
2.2. Were carers and people delivering the interventions aware of participants' assigned intervention during the trial?		PN

2.3. If Y/PY/NI to 2.1 or 2.2: Were there deviations from the intended intervention that arose because of the trial context?	-	-
2.4 If Y/PY to 2.3: Were these deviations likely to have affected the outcome?	-	-
2.5. If Y/PY/NI to 2.4: Were these deviations from intended intervention balanced between groups?	-	-
2.6 Was an appropriate analysis used to estimate the effect of assignment to intervention?	-	-
2.7 If N/PN/NI to 2.6: Was there potential for a substantial impact (on the result) of the failure to analyse participants in the group to which they were randomized?	-	-
Risk-of-bias judgement	Low risk	

Domain 3: Missing outcome data

Signalling questions	Comments	Response options
3.1 Were data for this outcome available for all, or nearly all, participants randomized?	Y	Y
3.2 If N/PN/NI to 3.1: Is there evidence that the result was not biased by missing outcome data?	-	
3.3 If N/PN to 3.2: Could missingness in the outcome depend on its true value?	-	
3.4 If Y/PY/NI to 3.3: Is it likely that missingness in the outcome depended on its true value?		
Risk-of-bias judgement	Low risk	Low / High / Some concerns

Domain 4: Risk of bias in measurement of the outcome

Signalling questions	Comments	Response options
4.1 Was the method of measuring the outcome inappropriate?	N	N
4.2 Could measurement or ascertainment of the outcome have differed between intervention groups?	PN	PN
4.3 If N/PN/NI to 4.1 and 4.2: Were outcome assessors aware of the intervention received by study participants?	PN	PN
4.4 If Y/PY/NI to 4.3: Could assessment of the outcome have been influenced by knowledge of intervention received?	-	-
4.5 If Y/PY/NI to 4.4: Is it likely that assessment of the outcome was influenced by knowledge of intervention received?		-
Risk-of-bias judgement	Low risk	Low / High / Some concerns

Domain 5: Risk of bias in selection of the reported result

Signalling questions	Comments	Response options
5.1 Were the data that produced this result analysed in accordance with a pre-specified analysis plan that was finalized before unblinded outcome data were available for analysis?	Y	Y
Is the numerical result being assessed likely to have been selected, on the basis of the results, from...		
5.2. ... multiple eligible outcome measurements (e.g. scales, definitions, time points) within the outcome domain?	PN	Y / PY / PN / N / NI
5.3 ... multiple eligible analyses of the data?	PN	Y / PY / PN / N / NI

**Risk-of-bias judgement**	Low risk	Low / High / Some concerns

Overall risk of bias

**Risk-of-bias judgement**	Low risk	Low / High / Some concerns
